# Dual targeting PD-L1 and 4-1BB to overcome dendritic cell-mediated lenalidomide resistance in follicular lymphoma

**DOI:** 10.1038/s41392-024-02105-7

**Published:** 2025-01-20

**Authors:** Zhong Zheng, Jian-Biao Wang, Rui Sun, Nan Wang, Xiang-Qin Weng, Tian-Yuan Xu, Di Fu, Yan Feng, Peng-Peng Xu, Shu Cheng, Li Wang, Yan Zhao, Bin Qu, Chuan-Xin Huang, Wei-Li Zhao

**Affiliations:** 1 Shanghai Institute of Hematology, State Key Laboratory of Medical Genomics, National Research Center for Translational Medicine at Shanghai, Ruijin Hospital Affiliated to Shanghai Jiao Tong University School of Medicine, Shanghai, China; 2 Department of Laboratory Medicine, Shanghai RuiJin Hospital, Shanghai Jiao Tong University School of Medicine, Shanghai, China; 3 State Key Laboratory of Microbial Metabolism, School of Life Sciences and Biotechnology, Shanghai Jiao Tong University, Shanghai, China; 4 Department of Immunobiology and Microbiology, Shanghai Institute of Immunology, Shanghai Jiao Tong University School of Medicine, Shanghai, China; 5 Pôle de Recherches Sino-Français en Science du Vivant et Génomique, Laboratory of Molecular Pathology, Shanghai, China

**Keywords:** Cancer microenvironment, Cancer therapy

## Abstract

Immunomodulatory agent lenalidomide is effective in treating follicular lymphoma (FL). We conducted the first trial of immunotherapy rituximab plus lenalidomide in newly diagnosed FL in China (NCT03715309). One-hundred and fifteen patients were enrolled and treated with rituximab 375 mg/m^2^ intravenously on day 0 and lenalidomide 25 mg orally on day 1–10 for 6 cycles of induction treatment, as well as lenalidomide for 6 cycles and rituximab for 8 cycles of maintenance treatment. We found that inferior progression-free survival of the patients was significantly associated with elevated serum β2m and lymph node >6 cm, linking to decreased lymphoma cell autophagy and dendritic cell infiltration within the tumor microenvironment. PU.1 transcriptionally downregulated PD-L1 (Programmed death ligand 1) expression and upregulated 4-1BBL (4-1BB ligand) expression, increased lymphoma cell autophagy and dendritic cell maturation via PD-1/PD-L1 and 4-1BB/4-1BBL interaction. In vitro in co-culture system and in vivo in murine xenograft model, knockdown of PU.1 induced lenalidomide resistance, but sensitized FL cells to bi-specific PD-L1/4-1BB antibody or combined treatment of PD-L1 inhibitor and 4-1BB agonist. Collectively, PU.1 is essential in immunomodulatory effect of FL through PD-1/PD-L1- and 4-1BB/4-1BBL-mediated microenvironmental modulation. Dual targeting PD-L1 and 4-1BB could be an alternative immunotherapeutic strategy in the chemo-free era of FL treatment.

## Introduction

Follicular lymphoma (FL) is the most common indolent non-Hodgkin lymphoma worldwide, comprising 15–25% of adult lymphomas with an incidence of 3–5/100,000 every year. Although FL was considered incurable, the use of anti-CD20 monoclonal antibody rituximab and the development of anthracycline-containing chemotherapy have significantly improved the outcomes for FL patients within the passing decades. Currently, the median survival of FL patients is nearly 20 years, but most patients experience recurrent disease relapse with increasing drug resistance. Meanwhile, a subset of patients has early progression or transformation, presenting a poor prognosis.^1^ Of note, rituximab plus immunochemotherapy (R-chemo) has considerable side effects, particularly hematological toxicity and non-hematological immunosuppression, gastrointestinal and cardiac events.^2^ As an alternative first-line immunochemotherapy, the primary follow-up data of RELEVANCE trial demonstrates that chemotherapy-free rituximab plus lenalidomide (R2) is applied to treat newly diagnosed FL with similar clinical efficacy.^3^ The second long-term follow-up data of RELEVANCE trial at 6 years continues to manifest that R2 has comparable, durable efficacy and safety versus R-chemo in newly diagnosed patients with FL.^3^ Multiple prognostic indexes have been developed for identifying patients at high risk of relapse or refractory, mainly as the Follicular Lymphoma International Prognostic Index (FLIPI) derived from patients receiving chemotherapy and the FLIPI2 derived from those receiving R-chemo.^4^ Besides, integrated models that combine clinical and molecular factors have also been proposed for patients receiving R-chemo, including m7-FLIPI, POD24-PI, and a 23-gene model. To date, no integrated models have been validated for patients receiving R2. Thus, it remains great interests to assess their utility in chemotherapy-free era of FL treatment.

The progression of FL manifests a high dependence on tumor microenvironment, which is composed of a varity of immune cells and stromal cells. In lymph nodes, the FL tumor microenvironment principally consists of CD4+ T regulatory cells (Tregs), CD4+ T follicular helper (Tfh) cells, CD8+ T cells, dendritic cells (DCs), macrophages, fibroblastic reticular cells (FRCs), and a smaller proportion of neutrophils and natural killer (NK) cells. Through soluble factors and ligand-receptor interactions, the crosstalk between tumor cells and tumor microenvironment cells facilitates tumor cell survival, induces immune escape, and also serves as promising prognostic biomarkers and potential therapeutic targets.^5^ Gene expression profile analysis showed that FL patients with low immune cell infiltration have higher FLIPI score and more POD24 (progression of disease within 24 months) than those with high immune cell infiltration.^6^ Recently, follicular helper T cells, Tregs, CD8+ T cells, macrophages, and DCs have been shown significantly associated with inferior survival in FL.^7^

Lenalidomide is an immunomodulatory agent that binds the cereblon E3 ubiquitin ligase complex and results in recruitment, ubiquitination, and degradation of transcription factors Aiolos and Ikaros, showing remarkable therapeutic efficacy in multiple B-cell neoplasms. Lenalidomide possesses direct anti-tumor activity, such as tumor apoptosis, cell cycle arrest and autophagy, as well as indirect effects mediated through multiple immune cells including T cells, macrophages, NK cells, DCs, and stromal cells within the tumor microenvironment.^8,9^ Beyond the direct cytotoxicity towards malignant B cells, recent studies have emphasized the therapeutic implications of lenalidomide through remodeling the interplay between malignant cells and immune cells. Lenalidomide has significant immune-mediated anti-lymphoma activity by increasing NK cell number and stimulating NK cell activation, resulting in increased immune synapse formation and cell cytotoxicity. Besides NK cells, lenalidomide lead to T cell proliferation and cytotoxicity. Moreover, lenalidomide upregulates MHC class I molecules and CD86 on DCs, promoting antigen uptake and antigen presentation of DCs for naive CD8+ T cells. The above findings provide evidence for the use of lenalidomide in combination with immunotherapies in TME-targeted treatment strategy.^10,11^

DC are the key antigen-presenting cells to provide costimulatory signals for T-cell priming and play an essential role for induction of anti-tumor immunity. Tumor cell autophagy could be triggered by DC through various mechanisms, including immune checkpoint modulation.^12,13^ Programmed death-1 (PD-1) and 4-1BB are predominantly expressed on DC, with respective ligand as programmed death ligand 1 (PD-L1) and 4-1BB ligand (4-1BBL) expressed on malignant B cells.^14^ PD-1/PD-L1 axis is critically involved in restraining DC survival and tumor growth,^15^ while 4-1BB/4-1BBL axis has important function in enhancing DC proliferation and anti-tumor immunity.^16^ Therefore, therapeutic targeting PD-1/PD-L1 and 4-1BB/4-1BBL interaction with downstream DC needs further investigation to explore potential immunotherapeutic approaches in FL.

In this study, we conducted the first clinical trial in China (NCT03715309) evaluating rituximab plus lenalidomide for newly diagnosed FL. Our findings identified DC as a critical microenvironmental factor contributing to disease progression in R2-treated FL patients. Furthermore, we explored the biological role and therapeutic implications of DCs in FL tumor microenvironment using both in vitro and in vivo models, providing clinical rational of dual targeting PD-L1 and 4-1BB in the chemo-free era of FL treatment.

## Results

### Elevated serum β2m and lymph node > 6 cm were associated with disease progression in FL upon treatment with rituximab plus lenalidomide

**Fig. 1:**
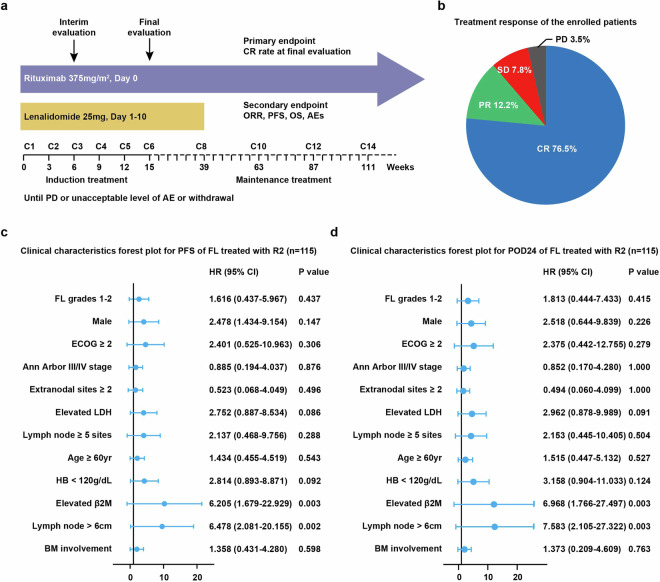
Elevated serum β2m and lymph node >6 cm were associated with disease progression in FL upon treatment with rituximab plus lenalidomide. **a** Study schema. Patients received rituximab 375 mg/m^2^ intravenously on day 0 and lenalidomide 25 mg orally on day 1–10 for 6 cycles of induction treatment, as well as lenalidomide for 6 cycles and rituximab for 8 cycles of maintenance treatment. **b** Treatment response of the enrolled patients. **c** Subgroup analysis of clinical parameters for PFS in R2-treated FL patients. **d** Subgroup analysis of clinical parameters for POD24 in R2-treated FL patients

A total of 115 patients with newly diagnosed FL were enrolled in a phase 2 trial of rituximab plus lenalidomide (NCT03715309) and referred to as the R2 cohort (Fig. 1a). Rituximab was dosed at 375 mg/m^2^ (day 0) and lenalidomide was dosed at 25 mg daily (day 1–10). Every 21 days was a cycle of treatment. All 115 subjects received the doses and treatment. Clinical and pathological characteristics of patients were summarized in Table 1. Briefly, 17.4% of the patients were older than 60 years, 56.5% were male, 85.2% had stage III/IV disease, and 16.5% had bulky disease. All 115 patients were assessed for treatment response, with 76.5% achieved complete remission (CR), 12.2% achieved partial remission (PR), 7.8% had stable disease (SD), and 3.5% had progression disease (PD) (Fig. 1b). Median time to best response was 2.5 months (range 2.0–4.1). Adverse events (AEs) of both hematological and non-hematological toxicity were listed in Table 2. For hematological AEs, grade 3–4 neutropenia and febrile neutropenia were found in 23 cases (20.0%) and 2 cases (1.74%), respectively. No grade 3–4 thrombocytopenia and grade 3–4 anemia were observed. For non-hematological AEs, grade 3–4 cutaneous reactions and tumor flare reactions were observed in 5 cases (4.35%) and in 1 case (0.09%), respectively. No grade 3–4 fatigue, nausea or vomiting, tumor lysis syndrome, and alopecia were presented (Table 2). With a median follow-up of 33.0 months (from 10.9 to 39.4), 2-year progression-free survival (PFS) was 89.6%, and 2-year overall survival (OS) was 99.1%.

**Table 1:** Baseline demographic and disease characteristics

Characteristics	R2 cohort
	(*n* = 115)
Median age (range)-year	53 (25–71)
Age ≥ 60 years-no (%)	20/115 (17.4%)
Male Sex	65/115 (56.5%)
ECOG Performance status-no (%)	
0	105/115 (91.3%)
1	10/115 (8.7%)
2	0/115 (0.00%)
Ann Arbor stage-no (%)	
I or II	17/115 (14.7%)
III or IV	98/115 (85.2%)
Bulky disease-no (%)	19/115 (16.5%)
Follicular lymphoma grade	
1 or 2	95/115 (82.6%)
3a	20/115 (17.4%)
Lactate dehydrogenase > ULN-no (%)	32/115 (27.8%)
Beta_2_-microglobulin > ULN-no (%)	63/115 (54.7%)

**Table 2:** Incidence of adverse events

	R2 (*N* = 115)	
	Any Grade	Grade 3 or 4
Hematological events		
Neutropenia	48 (41.7%)	23 (20.0%)
Anemia	12 (10.4%)	0 (0.00%)
Thrombocytopenia	5 (4.35%)	0 (0.00%)
Febrile neutropenia	4 (3.48%)	2 (1.74%)
Non-hematological events		
Fatigue	23 (20.0%)	0 (0.00%)
Cutaneous reactions	15 (13.0%)	5 (4.35%)
Nausea or vomiting	10 (8.70%)	0 (0.00%)
Tumor flare reaction	1 (0.09%)	1 (0.09%)
Tumor lysis syndrome	0 (0.00%)	0 (0.00%)
Alopecia	0 (0.00%)	0 (0.00%)

Forest plots were further performed to visualize the significance of major clinical factors (FL grades 1–2, Male, ECOG ≥ 2, Ann Arbor III/IV stage, extranodal involvement ≥ 2, Elevated LDH, Lymph node ≥ 5 sites, Age ≥ 60 years, HB < 120 g/dL, Elevated β2m, Lymph node >6 cm, and BM involvement) in terms of PFS. Elevated serum β2m and lymph node >6 cm were significantly associated with inferior PFS (*P* = 0.003 and *P* = 0.002, Fig. 1c) and POD24 (*P* both = 0.003, Fig. 1d). Thus, elevated serum β2m and lymph node >6 cm were highly predictive for disease progression in R2-treated FL patients.

### Altered dendritic cell maturation and function were induced by PU.1 and contributed to lenalidomide resistance in FL

**Fig. 2:**
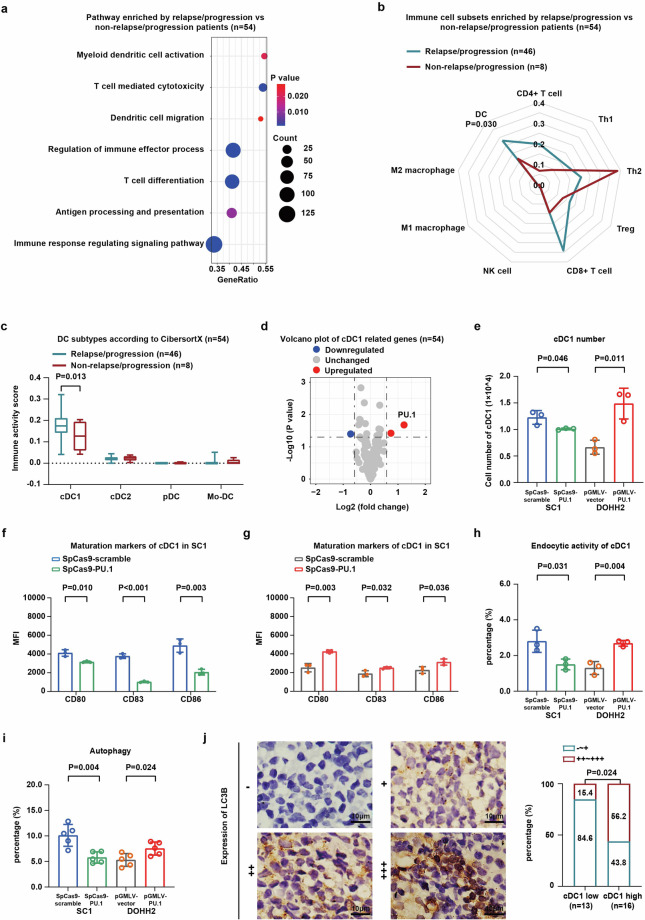
Altered dendritic cell maturation and function were induced by PU.1 and contributed to lenalidomide resistance in FL. **a** Gene ontology terms enriched in genes differentially expressed in samples with relapse/progression patients. The *P* values for the dysregulated pathways were represented by the color of the points, as determined by RNA sequencing. The point size indicates the gene number in the gene set. **b** Summary radar plot, with 0.05 radial intervals depicting normalized average scores of immune subsets in samples according to relapse/progression. **c** Deconvolution of bulk RNA-seq using CibersortX by the gene signatures defined in single-cell analysis depicting normalized average scores of DC subsets in samples according to relapse/progression. **d** Volcano plot of the distribution of all differentially expressed genes involved in cDC1. The cDC1 absolute cell numbers (**e**), maturation (**f**, **g**), and endocytic activity (**h**) detected by flow cytometry in samples of SpCas9-scramble transfected SC1 co-culture system and SpCas9-PU.1-transfected SC1 co-culture system, as well as pGMLV-vector transfected DOHH2 co-culture system and pGMLV-PU.1 transfected DOHH2 co-culture system. Mean with SD was presented (*n* = 3). **i** Quantification of typical autophagosomes observed by transmission electron microscopy was performed in the SpCas9-scramble and SpCas9-PU.1 transfected SC1 co-culture system, as well as the pGMLV-vector and pGMLV-PU.1 transfected DOHH2 co-culture system. Cells were counted from five randomly selected fields and analyzed statistically. Mean with SD was presented (*n* = 5). **j** Representative images of immunohistochemical analysis of autophagic cells in tumor samples from FL patients, comparing the cDC1 low group (*n* = 13) and cDC1 high group (*n* = 16)

To explore the underlying mechanism of lenalidomide resistance on FL progression, we performed RNA-sequencing analysis on available tumor samples of 54 patients collected before treatment. By differential gene expression analysis, we compared relapse/progression patients to non-relapse/progression patients (Fig. 2a). Immune-associated signaling pathways (dendritic cell and T cell-associated pathway) were the most enriched gene set in terms of relapse/progression. Meanwhile, immune activity scores of various immune cell types, including T-cell subsets (CD4+ T, Th1, Th2, Treg, and CD8+ T cells), NK cells, macrophages (M1 and M2 macrophages), and DC, were analyzed by Xcell. Relapse/progression patients presented lower DC than non-relapse/progression patients (*P* = 0.030, Fig. 2b). We further leveraged single-cell RNA sequencing data from tumor samples of FL patients to annotate plasmacytoid DC (pDC), conventional DC 1 (cDC1), conventional DC 2 (cDC2), and monocyte-derived DC (Mo-DC) subsets. Using the gene signatures defined in single-cell analysis, we performed an additional deconvolution of the bulk RNA-seq data using CibersortX, providing a more refined view of the DC subsets. A significantly decreased percentage of cDC1 was observed in the relapse/progression patients (*P* = 0.013, Fig. 2c). These data indicated the biological relevance of cDC1 within the tumor microenvironment in R2-treated FL patients. Immune activity scoring was then applied to differentiate the cDC1 high and the cDC1 low groups in FL. The median immune activity score for cDC1 was 0.023. Patients with immune activity scores equal to or above the median were classified into the cDC1 high group, while those below the median were classified as the cDC1 low group. cDC1-associated genes were differently expressed as plotted by volcano plot, with transcription factor PU.1 (SPI1) most significantly altered (Fig. 2d). To simulate the in vivo conditions of patients, we co-cultured FL cells with DCs. The gene expression and protein levels of PU.1 were evaluated by quantitative real-time PCR and Western blot in both SC1 and DOHH2 cells. PU.1 expression was higher in SC1 cells than in DOHH2 cells both at the transcriptional level and at the protein level (*P* = 0.008, Supplementary Fig. 1a, b). To determine the biological function of PU.1 on microenvironment cDC1, PU.1-knockdown plasmid and PU.1-overexpressing plasmid were established to regulate PU.1 expression in SC1 cells and DOHH2 cells. In the co-culture system, the absolute cell numbers of cDC1 cells decreased in the PU.1-knockdown SC1 co-culture system, whereas it increased in the PU.1-overexpressing DOHH2 co-culture system (*P* = 0.046 and *P* = 0.011, Fig. 2e). Apoptosis of cDC1 cells increased in PU.1-knockdown SC1 co-culture system, whereas it decreased on DOHH2 cells in PU.1-overexpressing DOHH2 co-culture system (*P* = 0.002 and *P* = 0.011, Supplementary Fig. 2a). Moreover, cDC1 maturation markers (CD80/CD83/CD86) were decreased in PU.1-knockdown SC1 co-culture system (*P* = 0.010, *P* < 0.001, and *P* = 0.003, Fig. 2f), while increased in PU.1-overexpressing DOHH2 co-culture system (*P* = 0.003, *P* = 0.032, and *P* = 0.036, Fig. 2g). The cDC1 exhaustion markers (PD-1/LAG3/TIGHT/VISTA) remained constant (Supplementary Fig. 2b, c).

In addition to cDC1 amount, cDC1 endocytic activity was decreased in PU.1-knockdown SC1 co-culture system, but increased in PU.1-overexpressing DOHH2 co-culture system (*P* = 0.031 and *P* = 0.004, Fig. 2h). No significant changes in lymphoma cell apoptosis or cell cycle were observed (Supplementary Fig. 2d–g). However, the expression of the autophagy marker LC3B was decreased in SC1 cells in the PU.1-knockdown SC1 co-culture system, while it was increased in DOHH2 cells in the PU.1-overexpressing DOHH2 co-culture system (*P* < 0.001 and *P* = 0.003, Supplementary Fig. 2h–j). Accordingly, ultrastructural analysis of lymphoma cells revealed a decrease in typical autophagosomes in SC1 cells, but an increase in DOHH2 cells (*P* = 0.004 and *P* = 0.024, Fig. 2i). Immunohistochemical analysis of tumor samples from FL patients revealed a higher frequency of LC3B-positive cells in cDC1 high group in comparison to cDC1 low group (*P* = 0.024, Fig. 2j). Together, altered cDC1 maturation and function were induced by PU.1, contributing to aberrant anti-tumor immunity and poor response to lenalidomide in FL patients.

### PU.1-mediated lenalidomide resistance through lymphoma cell PD-1/PD-L1 and 4-1BB/4-1BBL interaction with cDC1

**Fig. 3:**
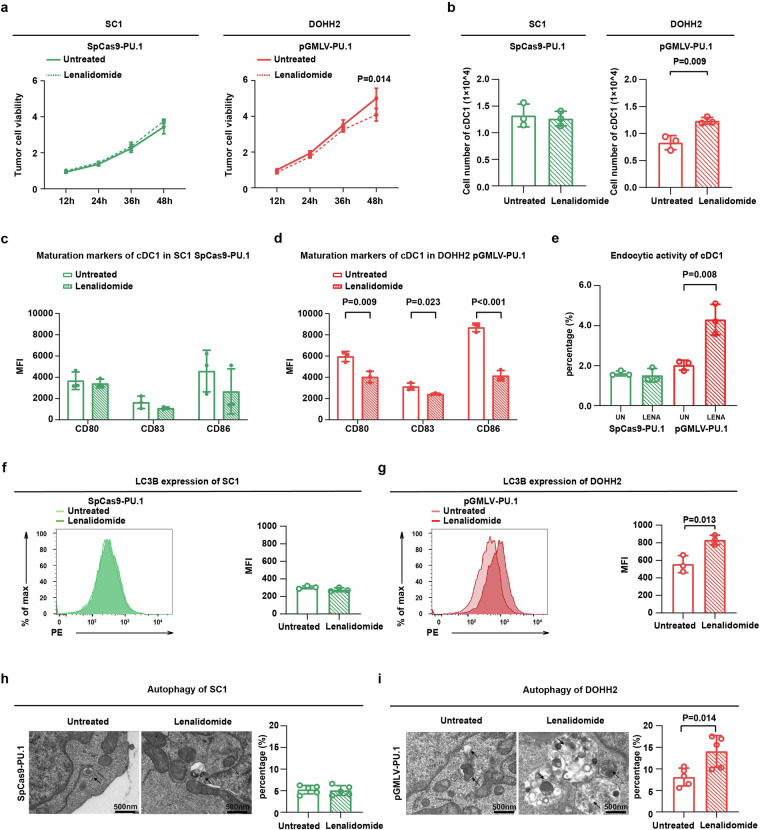
PU.1 mediated lenalidomide resistance in vitro. **a** Cell growth in SpCas9-PU.1-transfected SC1 (left) and pGMLV-PU.1-transfected DOHH2 (right) co-culture systems, treated with or without lenalidomide assessed by MTT assay. Mean with SD was presented (*n* = 5). **b** cDC1 absolute cell numbers in the SpCas9-PU.1 transfected SC1 co-culture system (left) and pGMLV-PU.1 transfected DOHH2 co-culture system (right) treated with or without lenalidomide assessed by flow cytometry. Mean with SD was presented (*n* = 3). cDC1 maturation in the SpCas9-PU.1 transfected SC1 co-culture system (**c**) and pGMLV-PU.1 transfected DOHH2 co-culture system (**d**) treated with or without lenalidomide assessed by flow cytometry. Mean with SD was presented (*n* = 3). **e** cDC1 endocytic activity in the SpCas9-PU.1 transfected SC1 co-culture system and pGMLV-PU.1 transfected DOHH2 co-culture system treated with or without lenalidomide assessed by flow cytometry. Mean with SD was presented (*n* = 3). LC3B expression in the SpCas9-PU.1 transfected SC1 co-culture system (**f**) and pGMLV-PU.1 transfected DOHH2 co-culture system (**g**) treated with or without lenalidomide assessed by flow cytometry. Mean with SD was presented (*n* = 3). Typical autophagosomes in the SpCas9-PU.1 transfected SC1 co-culture system (**h**) and pGMLV-PU.1 transfected DOHH2 co-culture system (**i**) treated with or without lenalidomide, were assessed using a transmission electron microscope. Five random fields were selected for cell counting and statistical analysis. Mean with SD was presented (*n* = 5)

Further experiments were conducted to investigate the functional role of PU.1 in lenalidomide resistance. PU.1-knockdown SC1 co-culture system and PU.1-overexpressing DOHH2 co-culture system were treated with lenalidomide. Comparing with the untreated group, no apparent change of tumor cell viability was observed in PU.1-knockdown SC1 co-culture system, while significant reduction of tumor cell viability was observed in PU.1-overexpressing DOHH2 co-culture system upon lenalidomide treatment (*P* = 0.014, Fig. 3a), confirming that PU.1 correlated with lenalidomide sensitivity. Accordingly, the absolute cell numbers of cDC1 remained constant in PU.1-knockdown SC1 co-culture system, but notably increased in PU.1-overexpressing DOHH2 co-culture system upon lenalidomide treatment (*P* = 0.009, Fig. 3b). Similar results were obtained on the maturation of cDC1 (Figs. 3c, d) and the endocytic activity of cDC1 (Fig. 3e). As for lymphoma cell autophagy, comparing with the untreated group, LC3B expression remained constant in PU.1-knockdown SC1 co-culture system (Fig. 3f), while increased in PU.1-overexpressing DOHH2 co-culture system upon lenalidomide treatment (*P* = 0.013, Fig. 3g), in consistent with amount of typical autophagosomes in lymphoma cells (Figs. 3h, i). Lymphoma cell response to lenalidomide, as well as cDC1 maturation and function, were not affected neither in SC1 cells nor in DOHH2 cells without PU.1 modulation (Supplementary Fig. 3). Therefore, PU.1 played an essential role in lenalidomide response though modulating cDC1 maturation and function in FL.

**Fig. 4:**
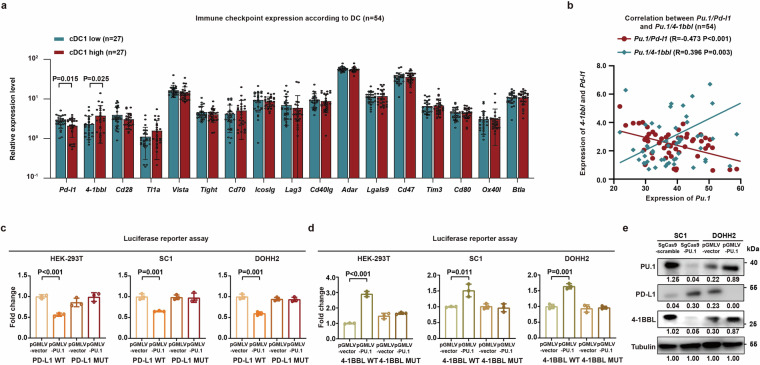
PU.1 modulated PD-1/PD-L1 and 4-1BB/4-1BBL-mediated FL cell interaction with cDC1. **a** Immune checkpoints with differential expression between the cDC1 high and low groups identified by RNA sequencing. **b** Correlation between PU.1 and PD-L1, as well as PU.1 and 4-1BBL expression calculated by Pearson correlation coefficient analysis. **c** Relative luciferase activities in PD-L1 WT or PD-L1 MUT transfected HEK-293T cells, SC1 cells, DOHH2 cells were detected. Mean with SD was presented (*n* = 3). **d** Relative luciferase activities in 4-1BBL WT or 4-1BBL MUT transfected HEK-293T cells, SC1 cells, DOHH2 cells were detected. Mean with SD was presented (*n* = 3). **e** Western blot of PU.1, PD-L1, and 4-1BBL expression in the SpCas9-PU.1 transfected SC1 cells and pGMLV-PU.1 transfected DOHH2 cells

Tumor expression of immune checkpoint genes was implicated in DC modulation, including *Pd-l1*, *4-1bbl*, *Cd28*, *Tl1a*, *Vista*, *Tight*, *Cd70*, *Icoslg*, *Lag3*, *Cd40lg*, *Adar*, *Lgals9*, *Cd47*, *Tim3*, *Cd80*, *Ox40l*, and *Btla*.^17^ Of these, *Pd-l1* and *4-1bbl* were the immune checkpoint genes exhibiting the greatest differential expression between the cDC1 high and cDC1 low groups in FL (*P* = 0.015 and *P* = 0.025, Fig. 4a). As calculated by Pearson correlation coefficient analysis, *Pu.1* displayed significant negative correlation with *Pd-l1* (*R* = −0.473, *P* < 0.001) and positive correlation with *4-1bbl* (*R* = 0.396, *P* = 0.003) (Fig. 4b). We then predicted potential binding regions of PD-L1 promotor (−1410 bp to −1416 bp) and 4-1BBL promotor (−1138 bp to −1148 bp) with PU.1 using bioinformatics analysis. Luciferase reporter assays demonstrated that PU.1 downregulated transcriptional activity in PD-L1 WT HEK-293T, SC1, and DOHH2 cells (*P* < 0.001, *P* = 0.001, and *P* = 0.001, Fig. 4c), an effect absent in PD-L1 MUT HEK-293T, SC1, and DOHH2 cells (Fig. 4c). Conversely, PU.1 upregulated the transcriptional level in 4-1BBL WT HEK-293T cells, SC1 cells and DOHH2 cells (*P* < 0.001, *P* = 0.011 and *P* = 0.001, Fig. 4d), an effect not observed in 4-1BBL MUT HEK-293T cells, SC1 cells and DOHH2 cells (Fig. 4d), indicating that PU.1 exerted its biological function via PD-L1 and 4-1BBL in FL cells. PD-L1 expression on SC1 cells was enhanced in PU.1-knockdown SC1 cells and inhibited in PU.1-overexpressing DOHH2 cells (Fig. 4e), while 4-1BBL expression on SC1 cells was inhibited in PU.1-knockdown SC1 cells, and enhanced in PU.1-overexpressing DOHH2 cells (Fig. 4e). Thus, PU.1 could regulate interaction between lymphoma cells and cDC1 through modulating PD-1/PD-L1 and 4-1BB/4-1BBL.

### Dual targeting PD-L1 and 4-1BB counteracted PU.1-mediated cDC1 alterations in vitro

**Fig. 5:**
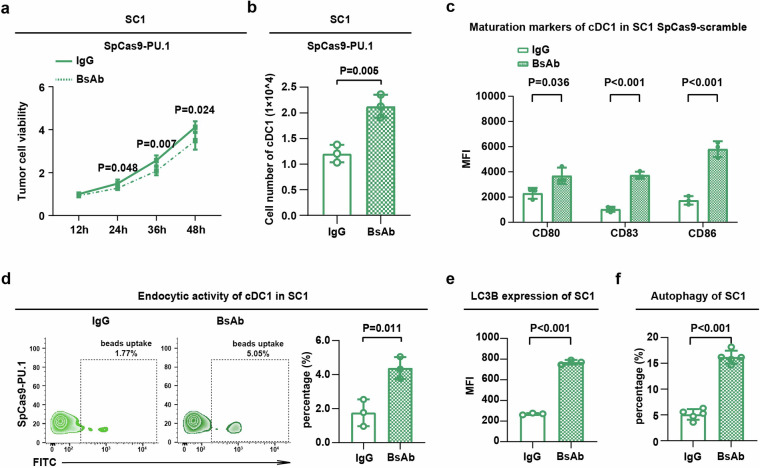
Dual targeting PD-L1 and 4-1BB counteracted PU.1-mediated cDC1 alterations in vitro*.* **a** Cell growth of the SpCas9-PU.1-transfected SC1 co-culture system, treated with or without the bi-specific PD-L1/4-1BB antibody assessed by MTT assay. Mean with SD was presented (*n* = 5). **b** cDC1 absolute cell numbers in the SpCas9-PU.1 transfected SC1 co-culture system treated with or without bi-specific PD-L1/4-1BB antibody assessed by flow cytometry. Mean with SD was presented (*n* = 3). **c** cDC1 maturation in the SpCas9-PU.1 transfected SC1 co-culture system treated with or without bi-specific PD-L1/4-1BB antibody assessed by flow cytometry. Mean with SD was presented (*n* = 3). **d** cDC1 endocytic activity in the SpCas9-PU.1 transfected SC1 co-culture system treated with or without bi-specific PD-L1/4-1BB antibody assessed by flow cytometry. Mean with SD was presented (*n* = 3). **e** LC3B expression in the SpCas9-PU.1 transfected SC1 co-culture system treated with or without bi-specific PD-L1/4-1BB antibody. Mean with SD was presented (*n* = 3). **f** Typical autophagosomes in the SpCas9-PU.1-transfected SC1 co-culture system, with or without bi-specific PD-L1/4-1BB antibody treatment assessed by transmission electron microscope. Cells were counted from randomly selected fields (*n* = 5). Mean with SD was presented

To explore the potential efficacy to overcome PU.1-mediated cDC1 alterations in FL, we treated the PU.1-knockdown SC1 co-culture system with bi-specific PD-L1/4-1BB antibody dual targeting PD-L1 and 4-1BB. PU.1-knockdown SC1 co-culture system was more sensitive to bi-specific PD-L1/4-1BB antibody treatment than the control IgG group (*P* = 0.048, *P* = 0.007, and *P* = 0.024, Fig. 5a). Comparing to the control IgG group, the absolute cell numbers of cDC1 were remarkably upregulated in the PU.1-knockdown SC1 co-culture system (*P* = 0.005, Fig. 5b), with maturation of cDC1 (*P* = 0.036, *P* < 0.001, and *P* < 0.001, Fig. 5c) and endocytic activity of cDC1 accordingly increased (*P* = 0.011, Fig. 5d) in the bi-specific PD-L1/4-1BB antibody group. LC3B expression and the presence of typical autophagosomes in lymphoma cells were commonly detected in the PU.1-knockdown SC1 co-culture system treated with the bi-specific PD-L1/4-1BB antibody (*P* both < 0.001, Figs. 5e, f). Therefore, PU.1-mediated cDC1 alterations and lymphoma cell autophagy could be counteracted by bi-specific PD-L1/4-1BB antibody in FL.

### Combined treatment of PD-L1 and 4-1BB antibody exhibited in vivo anti-lymphoma activity on PU.1-altered murine xenograft model

**Fig. 6:**
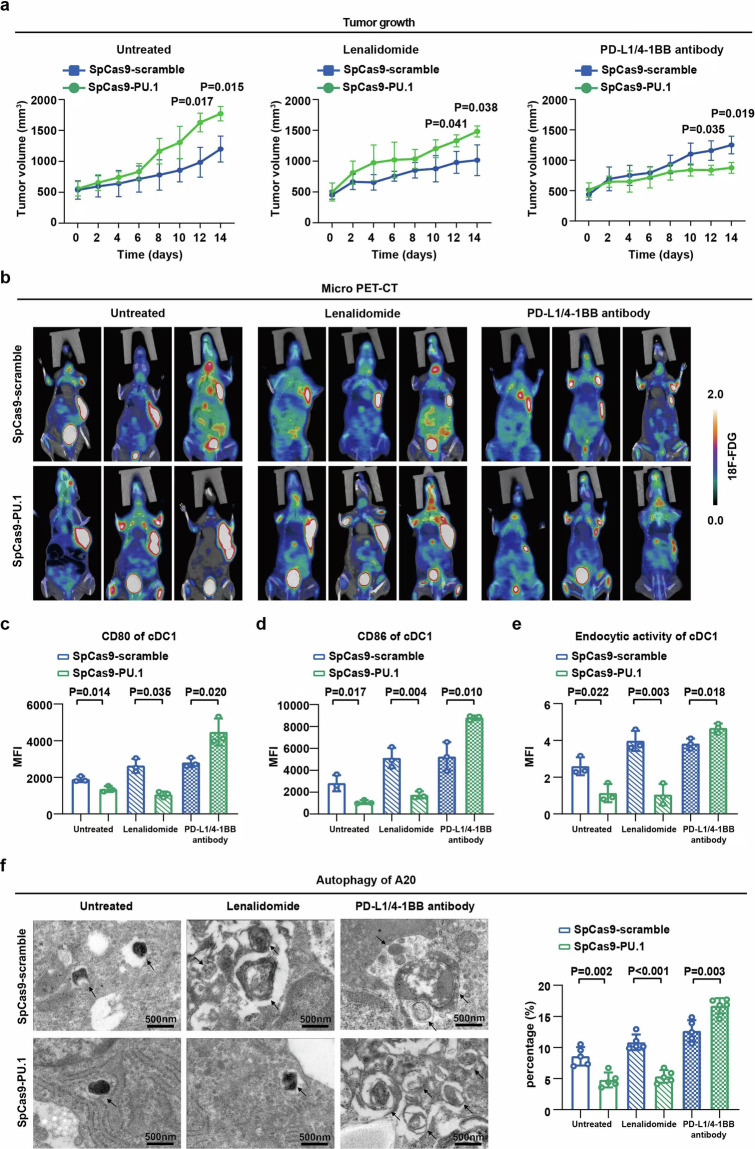
Combined treatment of PD-L1 and 4-1BB antibody exhibited in vivo anti-lymphoma activity on PU.1-altered murine xenograft model. **a** SpCas9-scramble and SpCas9-PU.1 cells were injected subcutaneously treated with or without lenalidomide, or co-treated with PD-L1 and 4-1BB antibody. Tumor size was measured. Mean with SD was presented (*n* = 3). **b** Standardized uptake value intensity in SpCas9-scramble group and SpCas9-PU.1 group treated with or without lenalidomide, or co-treated with PD-L1 and 4-1BB antibody assessed by Micro PET-CT. **c**, **d** cDC1 maturation in SpCas9-scramble group and SpCas9-PU.1 group treated with or without lenalidomide, or co-treated with PD-L1 and 4-1BB antibody assessed by flow cytometry. Mean with SD was presented (*n* = 3). **e** Flow cytometry analysis of cDC1 endocytic activity in SpCas9-scramble group and SpCas9-PU.1 group treated with or without lenalidomide, or co-treated with PD-L1 and 4-1BB antibody. Mean with SD was presented (*n* = 3). **f** Typical autophagosomes in the SpCas9-scramble group and SpCas9-PU.1 group, with or without lenalidomide treatment, or co-treated with PD-L1 and 4-1BB antibody assessed by transmission electron microscopy. Cells were counted from randomly selected fields (*n* = 5). Mean with SD was presented

A20 cells stably transfected with SpCas9-scramble or SpCas9-PU.1 were subcutaneously implanted to generate murine xenograft models. The sizes of SpCas9-PU.1 tumors were significantly larger than those of SpCas9-scramble tumors (*P* = 0.017 at day 12 and *P* = 0.015 at day 14, Fig. 6a left panel). Comparing with the lenalidomide group (*P* = 0.041 at day 12 and *P* = 0.038 at day 14, Fig. 6a middle panel), co-treatment of PD-L1 and 4-1BB antibody exhibited anti-lymphoma activity on SpCas9-PU.1 group (*P* = 0.035 at day 12 and *P* = 0.019 at day 14, Fig. 6a right panel). Small-animal PET/CT was used to image tumors (Fig. 6b). Consistent with in vitro study, maturation and endocytic activity of cDC1 were significantly decreased in the SpCas9-PU.1 group treated with or without lenalidomide treatment, which were increased by co-treatment of PD-L1 and 4-1BB antibody (Figs. 6c–e). Accordingly, comparing with the SpCas9-scramble group, autophagy in lymphoma cells was reduced in the SpCas9-PU.1 group treated with or without lenalidomide treatment (*P* = 0.002 and *P* < 0.001, Fig. 6f), whereas enhanced by co-treatment of PD-L1 and 4-1BB antibody (*P* = 0.003, Fig. 6f). Therefore, PU.1-altered B-lymphoma cells conferred to lenalidomide resistance, which could be overcome by combined treatment of PD-L1 and 4-1BB antibody through modulating PD-1/PD-L1 and 4-1BB/4-1BBL-mediated lymphoma cell interaction with DC.

## Discussion

Rituximab plus lenalidomide demonstrated as an effiective chemotherapy-free immunotherapy regimen with durable safety and was applied as front-line treatment of FL. In a phase II study comprising 66 patients with newly diagnosed FL, R2 treatment achieved promising efficacy, with the CR rate and overall response rate as 72% and 95%, and the 2-year PFS and OS were 86% and 100%.^18^ In another single-arm trial of R2 for newly diagnosed FL, 45 of 46 evaluable patients responded, with the CR rate as 87%, and the most common adverse events of grade 3 or higher were neutropenia.^19^ In a multicenter phase II trial of R2 led by the Alliance for Clinical Trials in Oncology, the CR rate (72%) and overall response rate (95%) were similarly high, and toxicities were comparable.^18^ These studies demonstrated that newly diagnosed FL patients are most likely to benefit from rituximab plus lenalidomide in the first line. Recent studies have demonstrated that TME components correlated with the survival of R2-treated FL patients. A high ratio of CD4+ T cells to CD8+ T cells, increased amounts of PD-1 positive tumor-infiltrating T cells, as well as signal regulatory protein alpha (SIRPα+) and colony stimulating factor-1 receptor (CSF1R+) macrophages were associated with inferior PFS, while higher amounts of GATA binding protein 3 (GATA3+) T helper were associated with good prognosis.^20,21^ Here, we first reported that clinical risk factors of elevated serum β2m and lymph node >6 cm translate to poor response to lenalidomide-containing immunotherapy. More importantly, we presented new insights into the complex microenvironment of FL showing that DC alterations have prognostic impact on lenalidomide resistance in FL.

Transcription factors may provoke lymphoma progression by regulating lymphoma cell interaction with the tumor microenvironment.^22,23^ PU.1 is a key communicator between FL cells and microenvironmental DC through immune checkpoint interaction.^24,25^ We demonstrated a significant correlation of PU.1 with expression of PD-L1 and 4-1BBL on lymphoma cells. Indeed, PU.1 downregulated PD-L1 and upregulated 4-1BBL, thereby initiated DC infiltration, and induced anti-tumor immunity in a PD-1/PD-L1 and 4-1BB/4-1BBL-dependent manner. DC can induce immune responses through presenting antigens to T cells and provide immunomodulatory signals through cell-cell interactions in solid tumors, including breast cancer, glioma, and renal cell carcinoma.^26^ Moreover, DC-based therapies allow a specific immune response associated with sustained lymphoma regression in FL.^27^ A phase I clinical trial evaluating the safety and clinical responses in relapse/refractory FL patients treated with monocyte-derived DC in combination with low doses of rituximab, demonstrating an overall response rate of 50%, and the remission is still ongoing in 2/4 of responding patients (median follow-up 26 months) with tolerable toxicities.^28^ Immune checkpoint modulators have revolutionized the treatment paradigm for multiple cancers, including lymphoma.^29^ In a single-arm phase II study comprising 30 patients with relapse/refractory FL, PD-1 antibody pembrolizumab combined with rituximab showed favorable efficacy, with the CR rate and overall response rate as 50% and 67%, the median PFS was 12.6 months.^30^ A first-in-human study of 4-1BB agonist antibody in combination with rituximab investigated demonstrated an overall response rate with 19.1% in 47 relapse/refractory FL patients.^31^ However, 4-1BB agonistic antibody is commonly associated with severe inflammatory liver toxicity.^32^ 4-1BB agonist antibody and PD-1 antibody could synergistically maximize anti-tumor immunity in ovarian cancer.^33^ Another phase Ib study comprising 23 patients with relapse/refractory solid tumor manifested that PD-1 antibody pembrolizumab combined with 4-1BB agonist antibody utomilumab showed promising efficacy, with six patients achieving CR or PR.^34^ ATG-101, a novel bi-specific PD-L1/4-1BB antibody, was designed to activate 4-1BB+ immune cells in a PD-L1-crosslinking dependent manner and effectively treat tumors without on-target-off-tumor liver toxicity.^35^ ATG-101 displayed potent antitumor activity in multiple solid tumor models, including immunologically “cold” tumor model, and tumor model with primary resistance to immune checkpoints inhibitors.^36^ Besides ATG101, multiple bi-specific PD-L1/4-1BB antibody have been developed and are being evaluated in clinical trials. GEN1046, another bi-specific PD-L1/4-1BB antibody, demonstrated encouraging single-agent activity in patients with solid tumors, with 65.6% of patients experiencing disease control. An analysis of the action mechanism of bi-specific PD-L1/4-1BB antibody significantly increased levels of cytokines such as interferon-gamma (IFNγ), C-X-C motif chemokine 10 (CXCL10). Furthermore, bi-specific PD-L1/4-1BB antibody could promote proportion of DC subsets, upregulate the expression of DC costimulatory molecules and DC activation markers, thus strengthening DC cell-T cell interaction, and generating a positive feedback loop of tumor microenvironment.^37^ Here, we provided evidence that lenalidomide resistance mediated by PU.1 could be counteracted by bi-specific PD-L1/4-1BB antibody, suggesting combination of immune checkpoint modulation as promising immunotherapy in FL.

Lenalidomide, as an immunomodulatory drug, exhibits a variety of important biological effects in DCs. In-vitro experiments of multiple myeloma demonstrated that lenalidomide promotes the differentiation of monocytes into immature DCs and the activation of mature DCs with increased endocytic capacity.^38^ Lenalidomide also enhances the expression of costimulatory molecules such as major histocompatibility complex class I and CD86 on DC, promotes the uptake of tumor antigens, and increases the efficiency of antigen presentation to CD8+ T cells in B-cell lymphoma.^39^ Lenalidomide also induces autophagy through recruitment of autophagy adapters and stabilization of autophagic structure.^40^ Lenalidomide resistance could attribute to decrement of autophagy, partly due to downregulation of E3 ligase expression.^41^ Moreover, DC-based cancer immunotherapies can induce strong immune response to eliminate tumor cells through autophagy.^42^ Immune checkpoints, such as PD-1/PD-L1 blockade and 4-1BB/4-1BBL stimulation, could act as novel autophagy regulators.^43,44^ Blocking the PD-1/PD-L1 axis via antibodies such as PD-1 antibody or PD-L1 antibody triggers autophagy in tumor cells, which allows the recycling of nutrients and the release of cytokines and extracellular vesicles that signal the ongoing damage in the malignant cells.^45^ Therefore, autophagy represents a biomarker of immunotherapy efficacy in FL.

In conclusion, PU.1 was related to lymphoma progression through modulating 4-1BB/4-1BBL and PD-1/PD-L1-mediated lymphoma cell interaction with DC, attributing to lenalidomide resistance in FL. Dual targeting PD-L1 and 4-1BB antibody could thus be promising in the chemo-free era of FL treatment.

## Materials and methods

### Patients

This was a phase 2 trial (NCT03715309), including patients aged 15–75 years who had been diagnosed with histologically proven FL (grade 1–3a). Pathological diagnosis was made according to the 2016 World Health Organization classification.^46^ Eligible patients should have an investigator-assessed diagnosis of stage II-IV. Patients should meet ≥1 of the following criteria to be considered “high” tumor burden according to the following GELF criteria: a tumor >7 cm in diameter; three nodes in three distinct areas each measuring >3 cm in diameter; symptomatic spleen enlargement; organ compression; ascites or pleural effusion; presence of systemic symptoms; leukemic phase (>5.0 × 10^9^/L circulating malignant cells). Patients with primary central nervous system lymphoma, positive for the human immunodeficiency virus or hepatitis B virus, other active cancers, or significant illnesses that would interfere with research therapy were excluded. The intravenous administration of 375 mg/m^2^ rituximab was performed on day 0 of the induction phase, which lasted from cycle 1–6. Additionally, 25 mg of lenalidomide was given orally per day from day 1 to day 10 in every cycle. In the induction phase, a total of 6 cycles of R2 treatment was conducted.

After achieving complete remission or partial remission at the last induction phase, patients received rituximab maintenance quarterly for 2 years, lenalidomide maintenance at 25 mg from day 1 to day 10 every 21 days for six cycles, or until the disease progression. The evaluation of treatment response was conducted using an adjusted version of the Lugano 2014 criterion.^47^ Positron emission tomography-computed tomography (PET-CT) will be evaluated at baseline, after three cycles for interim evaluation and at the end of treatment for final evaluation. Neck, chest, abdomen, and pelvis CT scans were performed quarterly until 1 year, then every 6 months until 2 years, and every year thereafter. The safety will also be evaluated by number of participants with treatment-related adverse events as assessed by the Common Terminology Criteria for Adverse Events (CTCAE) version 4.0. The Shanghai Ruijin Hospital’s institutional review board and ethical committee gave their approval to the study (2018-169). All participants gave written informed consent in accordance with the Declaration of Helsinki.

### RNA-sequencing

Gene expression patterns were examined using RNA-sequencing on tumor tissues from 54 R2-treated FL patients. RNA-sequencing protocols were described as previously reported.^48–50^ Utilizing XCell (https://xcell.ucsf.edu/), the tumor microenvironment was examined. The default XCell (*N* = 64) signature was used. This signature set includes 489 gene signatures that map to 64 distinct cell types. The XCell workflow included filtering cancer genes, generating gene signatures, choosing the best signature, knowing raw score transformation parameters, finding the reference matrix for spillover compensation, calculating scores for a mixture, and significance assessment.^51^

### Tumor sample single-cell suspension preparation and single-cell RNA sequencing

Tumor biopsy was cut into ~1-cm pieces in 1.5 mL low binding centrifuge tube (Axygen) filled with 1 mL RPMI-1640 medium (Gibco) with 10% fetal bovine serum (Gibco). Tumor sample was transported immediately to the lab on wet ice and processed within 24 h. Tumor biopsy was gently transferred to a new low-binding centrifuge tube with 1 mL cold HBBS, then inverted the tube no more than 10 times. Tumor biopsy and dissociated small pieces were spun down at 200–250 × *g* for 2 min at 4 °C. Supernatant was discarded. Repeat 2 more times to rinse off culture medium. 1010 μL dissociation reagent (Shanghai Yuanqi Biomedical) was added to each tumor biopsy tube and incubated at 37 °C water bath, invert the tube 10 times every 5 min to dissociate the tumor tissue. The dissociation process was stopped by transferring the dissociated single-cell suspension to a new tube with 1 mL neutralization solution after 30–60 min, the dissociation tube was ringed with no more than 3 mL cold PBS and all solution was transferred to the single-cell suspension collection tube. Dissociated single cells pellet was collected after centrifugation at 350 × *g* for 5 min and resuspended in 3 mL cold PBS. The single-cell suspension was spun down again at 350 × *g* for 5 min and supernatant was discarded. 1 mL red blood cell lysis buffer (Shanghai Yuanqi Biomedical) was added to the tube to resuspend the cell pellet. After 5 min, 3 mL PBS was added to the tube. The nucleated cells were spun down at 350 × *g* for 5 min, supernatant was gently discarded, the nucleated cell pellet was washed twice with PBS the nucleated cell pellet was resuspended in 200 μL PBS. 5 μL single-cell suspension was used for cell counting using hemocytometer. The concentration of single-cell suspension was adjusted to 3 × 10^5^ cells/mL in PBS. 3 × 10^4^ cells were then loaded onto a microfluidic chip (Singleron Biotechnologies). The single-cell whole transcriptome library was constructed according to the manufacturer’s instructions (GEXSCOPE Single Cell Human V(D) J Kit, Singleron Biotechnologies). The libraries were sequenced on MGISEQ-2000 instrument with 150 bp paired-end reads (MGISEQ-2000RS High-throughput Sequencing Kit, MGI Technology). The scRNA-seq library was sequenced for data of at least 100G.

### Data processing and cell type annotation

Single-cell RNA sequencing data from 5 FL samples were processed and aligned to the reference genome GRCh38-2020 using celescope version 1.15.0. Stringent quality filters were applied to remove cells with (1) number of detected genes (nFeature_RNA) < 200, (2) mitochondrial gene percentage (percent_mito) > 10%, and (3) ribosomal gene percentage (percent_ribo) < 5%. After filtering, the dataset included 30,095 genes detected across 45,781 cells. The filtered data were imported into Seurat (version 4.3.0), and normalization was performed using SCTransform. Principal component analysis (PCA) was used for dimensionality reduction. To remove batch effects, Harmony was applied after PCA to integrate the data across different samples. Specifically, the first 30 principal components were used as input to Harmony, which was run to align the cells in a shared low-dimensional space while preserving the biological variance. The Harmony-corrected embeddings were then used for subsequent clustering and visualization steps, including FindNeighbors, FindClusters, and UMAP. For cell type annotation, multiple well-established cell type-specific markers were used to annotate each cluster. Cell subtypes were further distinguished by assessing the expression of known markers along with the top differentially expressed genes (DEGs) defining each cluster. To assess differential expression, the FindMarkers function in Seurat was employed with the default Wilcoxon rank-sum test. Differentially expressed gene lists were refined using a threshold of corrected *p*-value (p_val_adj < 0.05) and an absolute Log2(Fold Change) greater than 0.25. Cells identified as myeloid cells were extracted, and the above steps were repeated to further analyze these cells. The extracted myeloid cells were reprocessed using SCTransform normalization, PCA for dimensionality reduction, and clustering using FindNeighbors and FindClusters functions. UMAP embeddings were recalculated using the first 20 PCA dimensions. Different DC clusters and other myeloid cell types were defined by evaluating the expression of well-known markers and the top DEGs defining each cluster.

### Bulk RNA deconvolution using CIBERSORTx

To generate a signature matrix for each cell subtype, we began by randomly selecting 500 cells from each identified subtype within our single-cell RNA-seq dataset. This selection ensured that the signature matrix captured representative transcriptional profiles across all relevant subtypes. The chosen cells were used as input for CIBERSORTx a computational tool used to impute gene expression sigantures and estimate the abundance of various cell types within a mixed cell population, based on gene expression data.^52^ Once the signature matrix was generated, we applied it to the bulk RNA-seq data from FL samples. We used the TPM data as input for CIBERSORTx to perform deconvolution analysis. The goal of this analysis was to estimate the proportion of each cell subtype present in the bulk samples, allowing us to impute cell fractions based on their transcriptional signatures.

### Cell lines and reagents

The human FL cell line SC1 and DOHH2 were purchased from DSMZ Cell Lines Bank (Braunschweig, Germany). HEK-293T and murine B-lymphoma cell line A20 were from American Type Culture Collection (Manassas, VA, USA). At 37 °C, **i**n an environment with 5% CO_**2**_, SC1, DOHH2, HEK-293T and A20 were cultured. Separation via Ficoll density gradient was used to obtain peripheral blood mononuclear cells (PBMCs) from a healthy donor, and the EasySepTM Human Pan-DC Pre-Enrichment Kit was used to sort DC cells. Selleck provided lenalidomide (Houston, TX, USA). A novel bi-specific PD-L1/4-1BB antibody ATG-101 and control IgG were obtained from Antengene (Shanghai, China).

### In vitro co-culture system

The co-culture experiment was conducted using Transwell cell culture chambers. The ratio of DC to lymphoma cells was identified as the effector (E) to target (T) ratio, which was 1:1, as indicated earlier.^53^ DC were positioned in the lower chamber and lymphoma cells were positioned in the upper chamber. Following the collection of cells from the lower chamber, the EasySepTM Human CD20+ Cell Isolation Kit was used to sort lymphoma cells.

### Lentivirus packaging and transfection

To knockdown PU.1 in SC1 cells, purified plasmid SpCas9-scramble or SpCas9-PU.1 was constructed. To overexpress PU.1 in DOHH2 cells, purified plasmid pGMLV-vector or pGMLV-PU.1 was constructed. Using lipofectamine 2000 (Invitrogen), HEK-293T cells were transfected with the plasmids mentioned above. The virus content in the HEK-293T cell culture supernatant was estimated to be around 3 × 10^8^ transducing units/ml. The SC1 or DOHH2 cells were treated with the lentiviral vector particles for an entire night. After treating the transfected cells with 4 μg/ml puromycin, the cells were separated, and stable clones were chosen using 1 μg/ml puromycin. Using lipofectamine 2000 (Invitrogen), Purified plasmids SpCas9-scramble or SpCas9-PU.1 were transfected into HEK-293T in order to knockdown PU.1 in A20 cells. The virus content in the HEK-293T cell culture supernatant was estimated to be around 3 × 10^8^ units/ml. The A20 cells were cultured with the lentiviral particles for an entire night. After 4 μg/ml puromycin treatment, the stably transfected cells were obtained, and stable clones were chosen after 1 μg/ml puromycin treatment.

### Luciferase report assay

The promoter region of 4-1BBL (−1138 to −1148 bp) was amplified from HEK-293T total cDNA using the following primers: forward 5′-GATAGGTACCGAGCTCTTACGCGTGGATTACAGGCGTGAGCCACTG-3′ and reverse 5′-CTTACTTAGATCGCAGATCTCGAGCAACCACCCTCACTCCTGGAG-3′. The XbaI restriction enzyme site was utilized. HEK-293T, SC1, and DOHH2 cells were co-transfected using Lipofectamine 2000 with luciferase reporter constructs, including pGMLV-vector or pGMLV-PU.1, along with 4-1BBL wild-type (4-1BBL WT) or mutant (4-1BBL MUT) constructs, and a luciferase reporter (10 ng/ml). The promoter region of PD-L1 (−1410 to −1416 bp) was similarly amplified from HEK-293T total cDNA using the forward primer 5′-GGTACCGAGCTCTTACGCGTAGC-3′ and the reverse primer 5′-GATCGCAGATCTCGAGCTGCA-3′, incorporating Mlul and XboI restriction enzyme sites. Using lipofectamine 2000, HEK-293T, SC1, and DOHH2 cells were co-transfected with luciferase reporter constructs expressing either pGMLV-vector or pGMLV-PU.1, luciferase reporter, either PD-L1 wild-type (PD-L1 WT) or PD-L1 mutant (PD-L1 MUT). The luciferase report assay was measured following the manufacturer’s protocol.

### Quantitative real-time PCR

Using Trizol reagent, total RNA from SC1 and DOHH2 cells was extracted. RNA was then used for reverse transcribing. SYBR Premix Ex Taq TM II (RR820A, TaKaRa) was used for quantitative real-time PCR in accordance with instructions provided by the manufacturer. *Pu.1* forward primer: 5′-GTGCCCTATGACACGGATCTA-3′; reverse primer: 5′- AGTCCCAGTAATGGTCGCTAT-3′. GAPDH forward primer of: 5′-GGAGCGAGATCCCTCCAAAAT-3′; reverse primer: 5′- GGCTGTTGTCATACTTCTCATGG-3′. The method of ^2−∆∆^CT was utilized to determine the relative value.

### Flow cytometry

Anti-HLA-DR, anti-CD11c, and anti-CD141 were used to detect cDC1. Anti-CD80, anti-CD83, and anti-CD86 were used to detect cDC1 maturation. Anti-PD-1, anti-LAG3, anti-TIGHT, and anti-VISTA were used to detect cDC1 exhaustion. Anti-LC3B was used to detect cell autophagy. The ANXA5/Annexin V-Phycoerythrin Kit was used to assess cell apoptosis Cell Cycle Kit was used to identify cell cycle. Flow cytometer was used to collect the data, and FlowJo software was used for further analysis.

### Western blot

Lysis buffer (Sigma Aldrich, Shanghai, China) was used for isolating and lysing SC1 and DOHH2 cells. Protein lysates (10 μg) were transferred to nitrocellulose membranes after being electrophoresed on a 10% SDS-PAGE. The membranes were then left to rock slowly at 4 °C for the entire night with PU.1 (2266S, Cell Signaling Technology), 4-1BBL (59127, Cell Signaling Technology), PD-L1 (ab213524, Abcam), and Tubulin primary antibody (5335, Cell Signaling Technology). The secondary detection was using an antibody coupled with horseradish peroxidase. With the use of chemiluminescence detection tool, immunocomplexes were visible.

### Immunohistochemistry

Using antibodies against LC3B (1:1000, 83506, Cell Signaling Technology), 5 μm-paraffin sections were applied following the instructions provided by the manufacturer. The expression levels of LC3B were assessed semi-quantitatively by calculating the proportion of positive cells as previously described.^54^ LC3B expression were calculated according to percentage of positive cells: −(≤10 punctate staining per cell), +(11–20 punctate staining per cell), ++(>20 punctate staining per cell without clustering) and +++(>20 punctate staining per cell with clustering).

### Transmission electron microscopy

Cell and tissue samples were fixed in 2% glutaraldehyde, washed in 0.1 M cacodylate, postfixed in 1% osmium tetroxide, and dehydrated using a series of ethanol. Ultrathin sections (60–100 nm) were placed on copper grids and analyzed with a transmission electron microscope (Philips CM120, Amsterdam, Netherlands). Cell autophagy was measured as previously indicated.^55^

### Murine xenograft model

Six-week-old BALB/c mice were provided by the Shanghai Laboratory Animal Center and their left flank was injected with 1 × 10^6^ A20 cells. Mice were kept on a regular diet in pathogen-free conditions with specific temperature (20–24 °C), humidity (40–60%), a 12-h cycle alternating between light and darkness., sterile food and water, regular bedding changes, good ventilation, and routine health monitoring to maintain a pathogen-free status. Treatments began on Day 0, when the tumor surface area was ~0.5 cm by 0.5 cm. For lenalidomide treatment, each mouse received an injection of 50 mg/kg lenalidomide via intraperitoneal for 2 weeks. For PD-L1 and 4-1BB antibody treatment, each mouse received an injection of 200 μg anti-mouse 4-1BB agonistic antibody (BE0296, Bioxcell, Lebanon, USA) and anti-mouse PD-L1 antibody (BE0101, Bioxcell) via the tail vein, three times each week for 2 weeks. The estimated tumor volumes were 0.5 × a(length) × b(width)^2^. The experimental protocols were approved by the animal ethics committee of Shanghai Ruijin Hospital and investigators followed the ethical code of animal use.

### Sample size and Statistical analysis

For sample size, we estimated that 73% of patients in the R2 group would achieve CR. One-hundred and fifteen patients will be required to show this difference with 5% significance (two-sided) and 90% power. The sample size was calculated by PASS software (NCSS, Kaysville, USA)

The assessment of progression-free survival (PFS) began on the day of therapy initiated and concluded on the day of disease progression or the final follow-up. The recognition of overall survival (OS) began on the day of diagnosis and concluded on the day of death or the final follow-up. Kaplan-Meier method was employed to construct survival analysis and compared by the log-rank test. The Chi-square test and Fisher’s exact test were utilized to compare categorical variables between several groups. Using the Cox regression method, univariate hazard estimates were calculated. Cox regression additionally required that the hazard ratio be proportional, which was calculated by Schoenfeld residual test. If the p-value was ≥0.05, then the hazard ratio was considered to be proportional. The in vitro results were reported as mean ± SD of three independent experiments and compared using the t-test. The Shapiro-Wilk test was used to verify normality prior to the t-test. The data was regularly distributed if the p-value was ≥0.05. The test for homogeneity of variance was Levene’s test. When the *p* value was ≥0.05, the assumption of equal variances was validated. The statistical software package SPSS version 20.0 was used for all statistical operations. Statistically significant was determined as *p* value less than 0.05.

## Supplementary information

supplementary

Study protocol

## Data Availability

The data in this study are available upon reasonable request to the corresponding author. Sequencing data can be viewed in GSA-human (https://ngdc.cncb.ac.cn/gsa-human) by pasting the accession number HRA003440 into the text search box or through the URL.

## Supplementary information

The online version contains supplementary material available at 10.1038/s41392-024-02105-7. Supplementary information accompanies the manuscript on the Signal Transduction and Targeted Therapy website http://www.nature.com/sigtrans.
