# Supplementary material for: Dual targeting PD-L1 and 4-1BB to overcome dendritic cell-mediated lenalidomide resistance in follicular lymphoma
Source: Signal Transduct Target Ther. 2025 Jan 20;10:29. doi: 10.1038/s41392-024-02105-7 (PMC11743790; doi:10.1038/s41392-024-02105-7)
Supplement: Supplementary file 1 — supplementary [file 41392_2024_2105_MOESM1_ESM.docx]

Supplementary Materials for

**Dual targeting PD-L1 and 4-1BB to overcome dendritic cell-mediated lenalidomide resistance in follicular lymphoma**

Zhong Zheng, Jian-Biao Wang, Rui Sun, Nan Wang, Xiang-Qin Weng, Tian-Yuan Xu, Di Fu, Yan Feng, Peng-Peng Xu, Shu Cheng, Li Wang, Yan Zhao, Bin Qu, Chuan-Xin Huang, and Wei-Li Zhao

Correspondence to: zhao.weili@yahoo.com

**This PDF file includes:**

Figures. S1 to S3


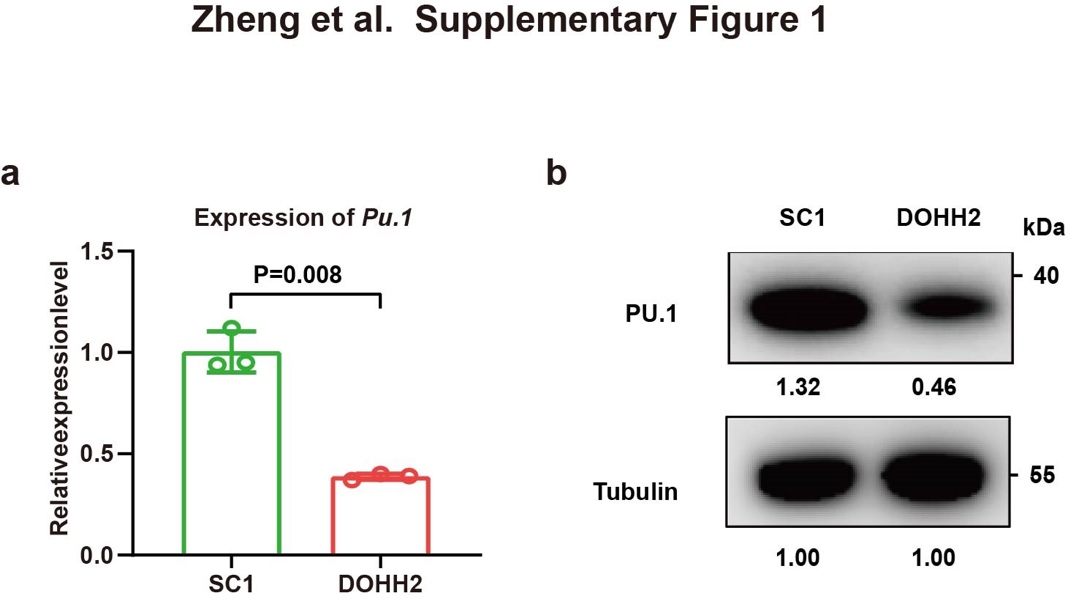


**Supplementary Figure 1.** **PU.1 expression in SC1 cells and DOHH2 cells.**

1. Real-time PCR analysis of PU.1 expression in SC1 cells and DOHH2 cells. Data are summarized as mean ± SD (n=3).
2. Western blot analysis of PU.1 in SC1 cells and DOHH2 cells


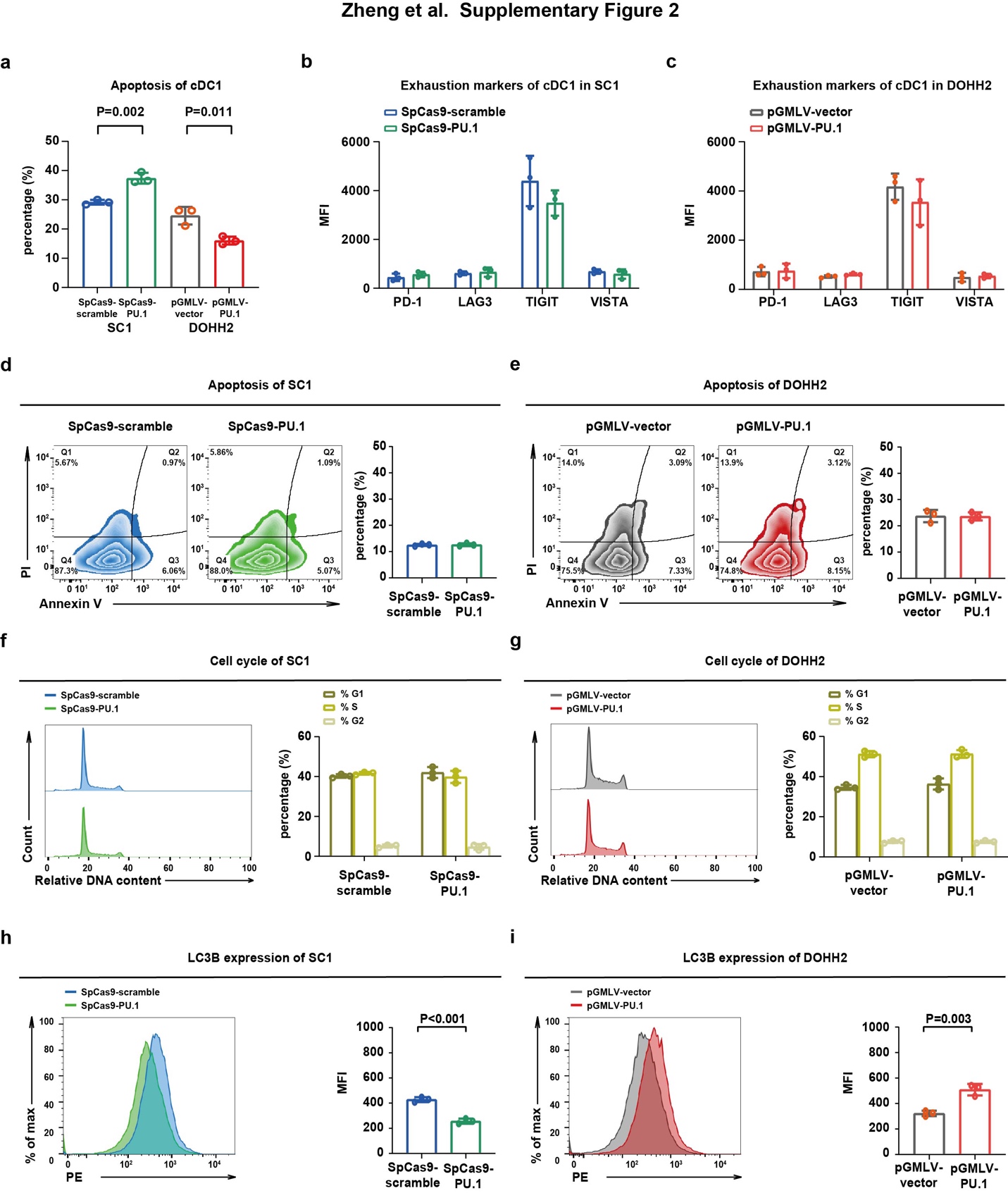


**Supplementary Figure 2. PU.1 mediated immune cell accumulation, tumor apoptosis, cell cycle and autophagy in FL.**

**(a)** Flow cytometry analysis of cDC1 apoptosis in the SpCas9-scramble transfected SC1 co-culture system and SpCas9-PU.1 transfected SC1 co-culture system, as well as pGMLV-vector transfected DOHH2 co-culture system and pGMLV-PU.1 transfected DOHH2 co-culture system. Data are summarized as mean ± SD (n=3).

**(b-c)** Flow cytometry analysis of exhaustion markers of cDC1 in the SpCas9-scramble transfected SC1 co-culture system and SpCas9-PU.1 transfected SC1 co-culture system (b), as well as pGMLV-vector transfected DOHH2 co-culture system and pGMLV-PU.1 transfected DOHH2 co-culture system (c). Data are summarized as mean ± SD (n=3).

**(d-e)** Flow cytometry analysis of cell apoptosis in the SpCas9-scramble transfected SC1 co-culture system and SpCas9-PU.1 transfected SC1 co-culture system (d), as well as pGMLV-vector transfected DOHH2 co-culture system and pGMLV-PU.1 transfected DOHH2 co-culture system (e). Data are summarized as mean ± SD (n=3).

**(f-g)** Flow cytometry analysis of cell cycle in the SpCas9-scramble transfected SC1 co-culture system and SpCas9-PU.1 transfected SC1 co-culture system (f), as well as pGMLV-vector transfected DOHH2 co-culture system and pGMLV-PU.1 transfected DOHH2 co-culture system (g). Data are summarized as mean ± SD (n=3).

**(h-i)** Flow cytometry analysis of LC3B expression in the SpCas9-scramble transfected SC1 co-culture system and SpCas9-PU.1 transfected SC1 co-culture system (h), as well as pGMLV-vector transfected DOHH2 co-culture system and pGMLV-PU.1 transfected DOHH2 co-culture system (i). Data are summarized as mean ± SD (n=3).

**
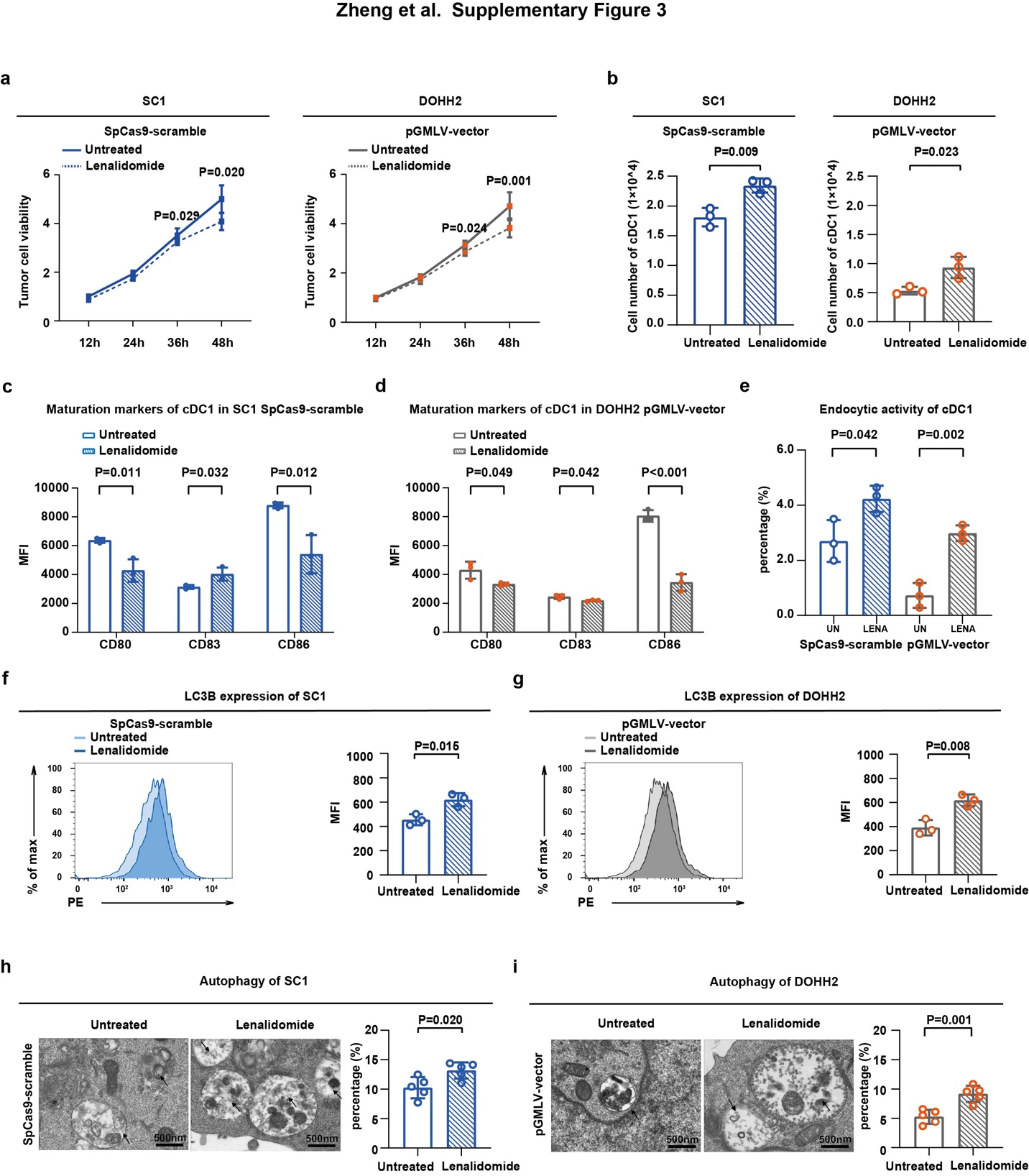
**

**Supplementary Figure 3. Lenalidomide mediated cDC1 alteration and tumor autophagy in vitro.**

**(a)** Cell growth in the SpCas9-scramble transfected SC1 co-culture system (left) and pGMLV-vector transfected DOHH2 co-culture system (right) treated with or without lenalidomide. MTT assay was adopted to measure cell viability. Data are summarized as mean ± SD (n=5).

**(b)** Flow cytometry analysis of cDC1 absolute cell numbers in the SpCas9-scramble transfected SC1 co-culture system (left) and pGMLV-vector transfected DOHH2 co-culture system (right) treated with or without lenalidomide. Data are summarized as mean ± SD (n=3).

**(c-d)** Flow cytometry analysis of cDC1 maturation in the SpCas9-scramble transfected SC1 co-culture system (c) and pGMLV-vector transfected DOHH2 co-culture system (d) treated with or without lenalidomide. Data are summarized as mean ± SD (n=3).

**(e)** Flow cytometry analysis of cDC1 endocytic activity in the SpCas9-scramble transfected SC1 co-culture system and pGMLV-vector transfected DOHH2 co-culture system treated with or without lenalidomide. Data are summarized as mean ± SD (n=3).

**(f-g)** Flow cytometry analysis of LC3B expression in the SpCas9-scramble transfected SC1 co-culture system (f) and pGMLV-vector transfected DOHH2 co-culture system (g) treated with or without lenalidomide. Data are summarized as mean ± SD (n=3).

**(h-i)** Transmission electron microscope showing typical autophagosomes in the SpCas9-scramble transfected SC1 co-culture system (h) and pGMLV-vector transfected DOHH2 co-culture system (i) treated with or without lenalidomide. The cells were counted from five visions selected at random and subjected for statistical analysis. Data are summarized as mean ± SD (n=5).
