# Supplementary material for: Dual targeting PD-L1 and 4-1BB to overcome dendritic cell-mediated lenalidomide resistance in follicular lymphoma
Source: Signal Transduct Target Ther. 2025 Jan 20;10:29. doi: 10.1038/s41392-024-02105-7 (PMC11743790; doi:10.1038/s41392-024-02105-7)
Supplement: Supplementary file 2 — Study protocol [file 41392_2024_2105_MOESM2_ESM.docx]

**Study Protocol**

**Protocol title:** A Phase 2, single-arm, phase 2 trial of rituximab plus lenalidomide in FL.

| Protocol number | NHL-R2 |
| --- | --- |
| Date: | 01 Sep, 2018 |
| Study Phase: | 2 |
| Study Design: | This is a phase 2 trial to evaluate the efficacy and safety of rituximab plus lenalidomide in patients with newly diagnosed FL. |
|  |  |

| **Synopsis** | |
| --- | --- |
| **Protocol Number:** NHL-R2 | **Study phase:** 2 |
| **Date of Protocol Synopsis:** 01 SEP, 2018 | |
| **Protocol Title:** A Phase 2, single-arm, phase 2 trial of rituximab plus lenalidomide in FL. | |
| **Objectives:**  **Primary objective:** The primary objective is to evaluate the complete response rate (CRR) at the end of treatment of rituximab plus lenalidomide in patients with newly diagnosed follicular lymphoma (FL).  **Secondary objectives:** The secondary objectives are to evaluate the complete response rate (ORR), progression free survival (PFS) and overall survival (OS). The safety will also be evaluated by number of participants with treatment-related adverse events as assessed by the Common Terminology Criteria for Adverse Events (CTCAE) version 4.0. | |
| **Study Sites:** Shanghai Rui Jin Hospital | |
| **Study Population:** Newly diagnosed patients with FL | |
| **Number of Subjects to be Enrolled:** 115 patients | |
| **Background:**  Non-Hodgkin lymphoma (NHL) is the indolent tumor originated in lymph nodes and other lymphoid tissues and the incidence is growing up at 3%-4% per year. Follicular lymphoma (FL) is a heterogeneous entity of non-Hodgkin lymphoma (NHL), accounting for 10%-20% of NHL. Rituximab, cyclophosphamide, doxorubicin, vincristine, and prednisolone (RCHOP) or RCHOP-like chemotherapy is widely used in FL. However, rituximab plus chemotherapy has considerable side effects, particularly hematological toxicity and non-hematological immunosuppression, gastrointestinal and cardiac events. Multiply combinational regimens have been attempted in FL, rituximab plus immunomodulatory agent lenalidomide (R2) achieve similar clinical efficacy as rituximab plus chemotherapy in newly diagnosed FL with less chemotherapy-associated side effects. We plan a phase 2, single-arm trial to evaluate the efficacy and safety of rituximab plus lenalidomide in a Chinese cohort of newly diagnosed patients with FL. | |
| **Diagnosis and Main Criteria for Inclusion/Exclusion:**  **Main Inclusion:**   - Newly diagnosed, histologically confirmed the following pathology subtype according to WHO 2008   classification: Follicular Lymphoma I-IIIA.   - ≥ 16 years of age. - Performance status of 2 or less. - Has no history of malignancy. - Has radiologically measurable disease. - Life expectancy ≥3months. - Voluntarily sign an informed consent. | |
| **Diagnosis and Main Criteria for Inclusion/Exclusion (Continued):**  **Main Exclusion:**   - Previous systemic chemotherapy or local therapy. - Has undergone hematopoietic stem-cell transplantation (HSCT). - Has previous treatment of lenalidomide. - Has an active infectious disease requiring general antibiotics, antifungal, or antiviral therapy. - Has an uncontrollable medical condition that might interfere with their participation in the study. - Echocardiography shows left ventricular ejection fraction (LVEF) ≤ 50%. - Woman in pregnancy or lactation. - Patient is known to be positive for Human immunodeficiency virus (HIV) infection. | |
| **Treatment** | |
| Study Group | Interventions |
| Rituximab plus lenalidomide Group | R2 (at each cycle):  Rituximab 375mg/m^2^, intravenously, Day 0  Lenalidomide 25mg, orally, Day 1-10  Every 21 days for 6 cycles |
| **Duration of Treatment:** A total of 6 cycles of treatment. | |
| **Criteria for Evaluation:**  **Efficacy:**  Positron emission tomography-computed tomography (PET-CT) will be evaluated at baseline, after three cycles for interim evaluation and at EOT for final evaluation. | |
| **Criteria for Evaluation (Continued):**  Responses will be assessed according to 2014 Lugano criteria for non-Hodgkin lymphoma. Central response assessment of PET and CT images will be performed by radiologists of Shanghai Ruijin Hospital, who are not informative of the treatment group. CT scans of the neck, thorax, abdomen, and pelvis will be repeated every three months thereafter to monitor disease progression in the first year, then every six months in the following two years, and yearly thereafter.  **Safety:**  The severity of adverse events will be assessed according to the CTCAE v4.0. | |
| **Statistical Plans:**  Statistical analysis will be conducted by or under the supervision of the study statistician. Statistical analysis will be performed using SPSS and GraphPad Prism.  The statistical analysis sets are defined as follows: The intention-to-treatment population (ITT) is defined as all subjects who were randomized to either of the treatment group and accepted at least one dose of treatment. ITT sets can be used for efficacy/safety analysis and demographic/baseline characteristics analysis.  The Fisher's exact test will be applied to categorical variables as appropriate. Analysis of covariance, or rank-sum test will be used, as appropriate, for continuous variables. Survival estimates will be calculated using Kaplan-Meier method. Log-rank test will be used to compare survival time between treatment groups. Univariate hazard estimates were generated with the Cox regression methods. Cox regression additionally required that the hazard ratio be proportional, which was calculated by Schoenfeld residual test. If the P value was ≥ 0.05, then the hazard ratio was considered proportional. Thereafter, variables with P value < 0.05 in univariate analyses were included in the multivariable sets.  The primary endpoint will be CRR at the end of treatment. Secondary endpoints will include ORR, PFS, OS and adverse events.  **Variable definition:**  Complete Response Rate (CRR): CRR was assessed by the investigators according to the Lugano 2014 criteria.  Objective response rate (ORR): ORR was defined as the proportion of patients with a complete response or partial response to treatment according to Lugano 2014 criteria.  Progression-Free Survival (PFS): PFS was defined as start date of treatment and ended with the date of disease progression or the last follow-up.  Overall Survival (OS): OS was recognized from start date of diagnosis and ended with the date of death or the last follow-up.  **Assumptions:**  We estimate that 73% of patients in the R2 group would achieve CR. One-hundred and fifty patients will be required to show this difference with 5% significance (two-sided) and 90% power. The number of patients achieving CR at the end of study will be reported by treatment group with 95% CI using normal approximation. Sample size was calculated by PASS software (NCSS, Kaysville, UT).  **Data quality assurance:**  To ensure good quality data, investigators and study site personnel will be familiarized with the study protocol, study procedures and principles of GCP during the trial initiation visit. All the data will be placed into the local database via a data registry server managed by the data collection group.  All adverse clinical experiences, whether observed by the investigator or reported by the patient, will be recorded, with details about the duration and intensity of each episode, the action taken with respect to the test drug, and the patient’s outcome. The investigator will evaluate each adverse experience for its relationship to the test drug and for its seriousness. CTCAE term (AE description) and grade: The descriptions and grading scales found in the revised NCI Common Terminology Criteria for the CTCAE v4.0. will be utilized for AE reporting. Concomitant medications will be coded using WHO Drug Reference List.  **Handling of missing data:**  Every effort will be made to contact patients who fail to return for scheduled visits. A patient is considered lost to follow-up if no information has been obtained by investigator for one year and at least three unsuccessful documented attempts of contact are available in source documentation. Missing or incomplete data for survival are managed by censored data analyses.  **Follow-up plan:**  Follow-up period will start at the end of treatment or at treatment discontinuation. Patient will be followed every 3 months for the first year and every 6 months up to end of follow-up period.  Follow-up assessments include:  • Physical examination including ECOG PS  • Hematology laboratory evaluations (RBC count, hemoglobin, hematocrit, WBC count and ANC, and platelet count)  • CT scans every 3 months for the first year and every 6 months for the second year and then every year until disease progression/relapse or end of follow-up period. | |
